# PV Interneurons: Critical Regulators of E/I Balance for Prefrontal Cortex-Dependent Behavior and Psychiatric Disorders

**DOI:** 10.3389/fncir.2018.00037

**Published:** 2018-05-16

**Authors:** Brielle R. Ferguson, Wen-Jun Gao

**Affiliations:** ^1^Department of Neurobiology and Anatomy, College of Medicine, Drexel University, Philadelphia, PA, United States; ^2^Department of Neurology and Neurological Sciences, School of Medicine, Stanford University, Stanford, CA, United States

**Keywords:** excitation/inhibition balance, GABA, PV interneurons, prefrontal cortex, cognition, psychiatric disorders

## Abstract

Elucidating the prefrontal cortical microcircuit has been challenging, given its role in multiple complex behaviors, including working memory, cognitive flexibility, attention, social interaction and emotional regulation. Additionally, previous methodological limitations made it difficult to parse out the contribution of certain neuronal subpopulations in refining cortical representations. However, growing evidence supports a fundamental role of fast-spiking parvalbumin (PV) GABAergic interneurons in regulating pyramidal neuron activity to drive appropriate behavioral responses. Further, their function is heavily diminished in the prefrontal cortex (PFC) in numerous psychiatric diseases, including schizophrenia and autism. Previous research has demonstrated the importance of the optimal balance of excitation and inhibition (E/I) in cortical circuits in maintaining the efficiency of cortical information processing. Although we are still unraveling the mechanisms of information representation in the PFC, the E/I balance seems to be crucial, as pharmacological, chemogenetic and optogenetic approaches for disrupting E/I balance induce impairments in a range of PFC-dependent behaviors. In this review, we will explore two key hypotheses. First, PV interneurons are powerful regulators of E/I balance in the PFC, and help optimize the representation and processing of supramodal information in PFC. Second, diminishing the function of PV interneurons is sufficient to generate an elaborate symptom sequelae corresponding to those observed in a range of psychiatric diseases. Then, using this framework, we will speculate on whether this circuitry could represent a platform for the development of therapeutic interventions in disorders of PFC function.

## Introduction

Cognitive impairment stifles independance by making even the simplest everyday tasks seem challenging and burdensome. Through limiting one’s ability to focus, encode, retain and manipulate information to make menial decisions, simple endeavors like traveling from one place to another, or completing a typical work assignment become arduous. This decreases productivity, and forces afflicted individuals to rely partially or wholly on the care of others. For most, this loss of independance can be crippling to one’s morale, resulting in increasing isolation from family, friends and society. Not surprisingly, disorders with reduced cognitive ability share high comorbidity with decreased sociability, as well as anxiety and depression. What remains unclear is whether these behaviors rely on a common circuitry, and if that circuitry could represent a platform for development of therapeutic interventions.

A large body of research suggests that the prefrontal cortex (PFC) is critical in regulating these behaviors and that the balance between excitatory and inhibitory neurotransmission (E/I balance) plays a fundamental role. The PFC is a unique associative cortical region that receives multimodal inputs from sensory regions, limbic structures and neuromodulatory nuclei (Carmichael and Price, 1996; Hoover and Vertes, 2007), and is taxed with processing these heterogeneous inputs to effectively guide ongoing behavior. The seamless execution of a range of behaviors relies on the integration of past experiences and current goals to select appropriate behavioral programs. Across species, we deem the myriad abilities linked to the PFC as “executive functioning,” and this category includes working memory, cognitive flexibility, planning, error-monitoring, decision-making, attention and social cognition. Although we have yet to unravel the precise mechanisms of information representation and processing in the PFC, recent optogenetic and imaging breakthroughs along with decades of lesion, pharmacological, and electrophysiological data highlight certain fundamental principles that may have relevance across PFC-dependent functions. Here, we argue that the E/I balance is essential for the proper execution of a range of PFC-dependent behaviors, and targeting this balance may be an effective approach in disorders that harbor related behavioral impairments.

We will begin with a discussion of GABAergic signaling and E/I balance using foundational research from sensory cortices to inform our interpretation of data from the PFC. Then, we will summarize data supporting our hypotheses, with a primary focus on research in rodents, as currently these types of studies allow for the most experimental flexibility and direct manipulation of specific neuronal types that regulate E/I balance. We acknowledge that the agranular rodent frontal cortex has no apparent structural equivalent of the dorsolateral PFC (dlPFC), a region in humans and primates linked to many of the behaviors that will be discussed in this review. Additionally, the human and non-human primate PFC are substantially more developed and complex relative to the comparable region in the rodent. Nevertheless, using a combination of tracing studies and lesion-symptom mapping, many scientists have concluded that the prelimbic region of the rodent medial PFC (mPFC) serves as the closest functional homolog to the dlPFC in humans and non-human primates (Uylings et al., 2003; Seamans et al., 2008). Human and primate studies will be utilized for comparison and framing purposes, but a detailed review of data from all three species is beyond the scope of this review. For a discussion involving a more comprehensive overview of clinical research discussing E/I balance and implications for psychiatric disease, see the following recent reviews (Foss-Feig et al., 2017; Krystal et al., 2017; Takarae and Sweeney, 2017).

## GABAergic Signaling and E/I Balance

Within the cortex, there are two primary types of neurons: glutamatergic excitatory pyramidal neurons and GABAergic inhibitory interneurons. While a network of purely excitatory connections offers little computational complexity, GABAergic interneurons confer a circuit with tremendous flexibility, dynamically modulating the gain of pyramidal neuron responses from simple all-or-none responsiveness (Klausberger et al., 2003; Isaacson and Scanziani, 2011). Through inhibition of neighboring pyramidal neurons, GABAergic interneurons act as an important brake on excitatory signaling, control spike generation and timing (Pouille and Scanziani, 2001), and prevent incoming excitation from afferent structures from causing runaway feed-forward excitation (Dichter and Ayala, 1987).

GABAergic interneurons are a diverse family that can be classified by their morphology, electrophysiological properties, or histological markers (Markram et al., 2004, 2015). The most common nomenclature segregates interneurons into three broad types, parvalbumin (PV), somatostatin (SST) and ionotropic serotonin receptor 5HT3a (5HT3aR) expressing interneurons (Rudy et al., 2011). Although not entirely distinct, this population minimizes overlap between the groups and accounts for nearly the entirety of known interneuronal subtypes.

The most common subtype, PV interneurons, are known for their fast-spiking phenotype, low input resistance, and high-amplitude rapid after-hyperpolarization (AHP; Kawaguchi et al., 1987; Kawaguchi and Kubota, 1997). This combination of properties confers an ability to fire a rapid train of action potentials unlike any other neuron in the cortex. PV interneurons can be further divided into two subtypes: basket cells that innervate the soma and proximal dendrites, and chandelier cells that synapse onto the axon initial segment (Kawaguchi and Kubota, 1997; Petilla Interneuron Nomenclature Group et al., 2008). Relatively less is known about the properties and function of chandelier cells, and data demonstrate their action may not be purely inhibitory at the axon initial segment (Szabadics et al., 2006). PV basket cells are more common, and by virtue of being easier to identify and study, their properties and role in circuit integration in the normal brain have been characterized in greater detail. Data from paired recordings indicate that a single PV interneuron contacts nearly every local pyramidal neuron providing PV basket cells with an unparalleled ability to regulate the activity of nearby pyramidal neurons (Packer and Yuste, 2011). This allows for a tremendous level of feedforward and feedback inhibition that serves several important functions (Hu et al., 2014).

An ability of PV interneurons that appears to be universal across cortical regions is controlling spike timing in neighboring excitatory neurons (Pouille and Scanziani, 2001; Wehr and Zador, 2003). Pyramidal neurons receive constant barrages of excitatory synaptic input or excitatory post-synaptic potentials (EPSPs), and are tasked with deciding whether or not to fire an action potential. Given the density of excitatory connections both from afferent structures and within the local circuit, along with the high level of synaptic divergence, excitation of a sufficient magnitude to generate an action potential will likely recruit a large portion of the cortical network, making it challenging to represent information with any specificity. This creates two computational dilemmas, creating a network state such that all excitatory neurons are not firing constantly, as well as allowing for distinct neurons or groups of neurons to have differential responsiveness to EPSPs. One issue is addressed by feedforward inhibition (FFI), a principle illustrated canonically in thalamocortical circuits. A major source of excitatory cortical input is the thalamus, whose neurons branch and synapse onto both excitatory and inhibitory neurons. Seminal physiological studies demonstrated that when thalamic fibers are electrically stimulated, this causes a direct excitatory response in cortical pyramidal neurons, followed shortly by a strong hyperpolarization due to the activation of neighboring PV basket cells (Agmon and Connors, 1991; Castro-Alamancos and Connors, 1997). This enhances the temporal fidelity of pyramidal neuron responsiveness by limiting the window in which incoming excitation can generate an action potential, and has also been observed in the mPFC (Cruikshank et al., 2012; Delevich et al., 2015). The second issue is mitigated through another property of PV interneuron activity, gain control, or “the rate at which the firing of a neuron increases in response to increasing excitatory input” (Isaacson and Scanziani, 2011). This transforms pyramidal neuron responsiveness from a simple go or no-go pattern of activity to having the capacity to represent a broad range of input levels in their firing patterns, a feature that is likely to be crucial in the mPFC, which processes complex multimodal information.

By innervating pyramidal neurons at the soma, PV basket cells are strategically positioned to exert both FFI and gain control. Additionally, PV basket cells have been implicated in the generation of gamma oscillations (30–80 Hz, Buzsáki and Draguhn, 2004), an oscillation range linked to cognition and information processing across species (Howard et al., 2003; Gaetz et al., 2011; Lundqvist et al., 2016). PV basket cells have the ability to fire at frequencies corresponding to gamma oscillations (Gulyás et al., 2010). These interneurons also synapse primarily onto alpha1-gamma-Aminobutyric acid receptors (α1-GABA_A_Rs) that are present at high levels on the soma (Fritschy and Mohler, 1995; Nusser et al., 1996), and have a decay constant corresponding to the rising phase of gamma waves (Gonzalez-Burgos and Lewis, 2008). Demonstrating a definitive link, a pair of studies utilized a battery of optogenetic tools to silence or activate cortical PV interneurons, and found them necessary and sufficient in the generation and maintenance of gamma oscillations (Cardin et al., 2009; Sohal et al., 2009). Increasing their activity not only amplified cortical gamma oscillations, but also enhanced information processing by increasing the gain of incoming information and improving behaviors dependent on the manipulated brain regions. Data also indicate the presence of significant gamma alterations in diseases with cognitive impairment, such as schizophrenia and autism spectrum disorders (ASD; Rojas and Wilson, 2014; McNally and McCarley, 2016).

In order for these processes to occur efficiently, levels of inhibition and excitation must remain in the appropriate balance, which in the mPFC has been measured as approximately 20/80% (excitation/inhibition; Le Roux et al., 2008; den Boon et al., 2015). This balance is not static and requires that inhibition be responsive to fluctuations in cortical state and levels of excitatory input (Galarreta and Hestrin, 1998; Shu et al., 2003; Atallah and Scanziani, 2009; Xue et al., 2014). PV interneurons are well suited for helping maintain the proper balance, as their activity is associated with “divisive” rather than subtractive inhibition, which would allow PV interneurons to respond proportionally to dynamic excitation within cortical circuits (El-Boustani and Sur, 2014). Within this balance, however, there must be the capacity for change or plasticity. Data suggests that E/I balance helps facilitate the induction of long-term potentiation, which could be critical in allowing the PFC to remain flexible and adapt to new stimulus-response contingencies (Staff and Spruston, 2003; Marder and Buonomano, 2004).

## Pharmacological Manipulation of E/I Balance and Behavior

Two cognitive abilities that are among the most consistently associated with damage to the PFC are working memory (Kolb et al., 1974; Stokes and Best, 1990; Kesner et al., 1996) and cognitive flexibility (Ragozzino et al., 1999; Birrell and Brown, 2000; Block et al., 2007; Bissonette et al., 2008). Working memory is the ability to maintain, manipulate and recall information to guide behavior (Baddeley, 1992; Goldman-Rakic, 1994), while cognitive flexibility involves the capacity to update strategies in order to obtain a reward (Bissonette et al., 2013). Both represent key building blocks of higher-level cognitive processes and are often severely impacted in the context of psychiatric disease. Social cognition is similar in denotation to working memory, but applies the principles of acquiring, storing, and manipulating information specifically to the context of interacting in a flexible and appropriate manner with conspecifics (Adolphs, 1999). Supporting its reliance on the frontal cortex are lesion data from humans and non-human primates indicating that damage to the ventromedial and orbital frontal cortex consistently result in abnormal social behavior (Ackerly and Benton, 1948; Butter and Snyder, 1972).

PFC infusion of bicuculline, a GABA_A_R antagonist, disrupts working memory and cognitive flexibility in multiple species (Sawaguchi et al., 1988, 1989; Enomoto et al., 2011; Paine et al., 2011; Auger and Floresco, 2015). *In vivo* multi-unit recordings in primates revealed that following bicuculline administration (which would increase the E/I balance), putative FS interneurons that were not linked to any specific task epochs of a spatial working memory task begin to show spatial tuning. Additionally, pyramidal neurons that once exhibited directional sensitivity, began to respond to their non-preferred directions, and neurons that had no task-modulated activity respond erroneously to random directions and task epochs (Rao et al., 2000). In concert, these activity alterations would greatly reduce the signal-to-noise ratio, severely limiting the ability of the circuit to generate and relay correct motor commands with precision. Circuits supporting normal patterns of social interaction may be regulated similarly, as bicuculline infusion into the rat mPFC reduces the number and duration of interactions as well as weakens the preference for a social stimulus compared to a non-social object (Cochran et al., 2002).

Conversely, studies that utilize enhancement of GABAergic transmission have been informative, as this approach would also disrupt the E/I balance. Muscimol, a GABA_A_ agonist, is used commonly as a means for transient inactivation of brain regions. Direct infusion in the mPFC in rodents has resulted in impairments in working memory (Urban et al., 2014), retention of strategy-switching behavior (Rich and Shapiro, 2007) and social play behavior (van Kerkhof et al., 2013, 2014).

## Optogenetics, Imaging and Physiology Highlight the Role of GABAergic Signaling in PFC-Dependent Behavior

Advances in tools available for bidirectional modulation of neuronal activity with cell-type specificity have expanded our understanding of the role of PV interneurons in regulating PFC E/I balance and PFC-dependent behaviors. Additionally, the development of genetically encoded calcium indicators, like gCaMP (Chen et al., 2013), allows researchers to monitor activity or calcium transients selectively in PV interneurons or other neuronal subtypes. With these technologies, researchers can catalog the entire population of mPFC interneurons from learning and strategy acquisition through successful performance. Many findings confirm and fine-tune long-standing hypotheses about how PV interneurons regulate pyramidal neuron activity, oscillatory dynamics, and cortical information representation, but allow for more detailed investigation of their causal role in behavior with enhanced temporal and cellular control.

One extremely useful feature of optogenetics (Deisseroth, 2011) has been the *in vivo* identification of different neuronal subtypes, or “optical tagging” (Zhao et al., 2011; Roux et al., 2014), allowing researchers to confirm the circuit consequences of PV interneuron activation as well as how these interneurons contribute to particular behaviors using recording or imaging. For example, optical tagging revealed that PV interneurons provide rapid synchronous inhibition of mPFC excitatory neurons in contrast with the more variable and less forceful inhibition provided by SST interneurons (Kvitsiani et al., 2013). A similar approach was used to demonstrate that PV interneurons increase their firing during goal-directed behavior. In this same study, researchers also showed that increases in PV activity were correlated with inhibition of certain pyramidal neurons and enhancement of others, potentially representing ensembles of relevant vs. irrelevant information for successful responding respectively (Kim H. et al., 2016). Increases in PV activity have also been implicated in the extinction of cue-based responding (Sparta et al., 2014), a process that may mediate rule-shifts during cognitive flexibility tasks.

During working memory assays, PV interneurons in the mPFC fire prominently throughout short delays (5 s or less) between sample and choice presentation (Kim D. et al., 2016), and show preferential firing toward go trials in go, no-go task (Kamigaki and Dan, 2017). However, more complicated tasks reveal they may encode information about distinct task epochs. Lagler et al. (2016) utilized a multidimensional delay task, where a differential gustatory cue signaled the location of reward in a Y-maze, and quantified PV interneuron activity during discrete phases of the assay. Interestingly, mPFC PV interneurons exhibited episodic modulation, and a large group showed preferential activation during the “goal run, ” when the mouse was proceeding through the base of the Y-maze before making a choice (Lagler et al., 2016). PV interneurons in the mPFC also increase their firing in response to typical social interactions, compared to the investigation of a non-social object (Selimbeyoglu et al., 2017).

Modulation of PV activity has also been useful in demonstrating one of the more obvious principles of E/I balance, that too much inhibition can also impair PFC circuit function. Optogenetic or pharmacogenetic activation of PV interneurons has been an effective method of disrupting the function of particular brain regions, suggesting that alterations in E/I balance in either direction can alter behavior. We recently demonstrated that increasing the excitability of mPFC PV interneurons (thereby reducing the E/I balance) significantly impairs working memory, cognitive flexibility and social interaction (Ferguson and Gao, 2018). Similarly, optogenetic activation of PV interneurons or GABAergic neurons more broadly disrupts delay-related activity and working memory performance (Rossi et al., 2012; Liu et al., 2014). These data corroborate pharmacological studies using muscimol, a GABA agonist, as a method of inactivating PFC to elucidate its role in various behaviors (Narayanan and Laubach, 2006; Urban et al., 2014). Correspondingly, working memory and social interaction deficits can be recapitulated by enhancing pyramidal neuron excitability (enhancing E/I balance; Yizhar et al., 2011; Liu et al., 2014; Kamigaki and Dan, 2017).

## Building the mPFC Microcircuit

**Figure 1:**
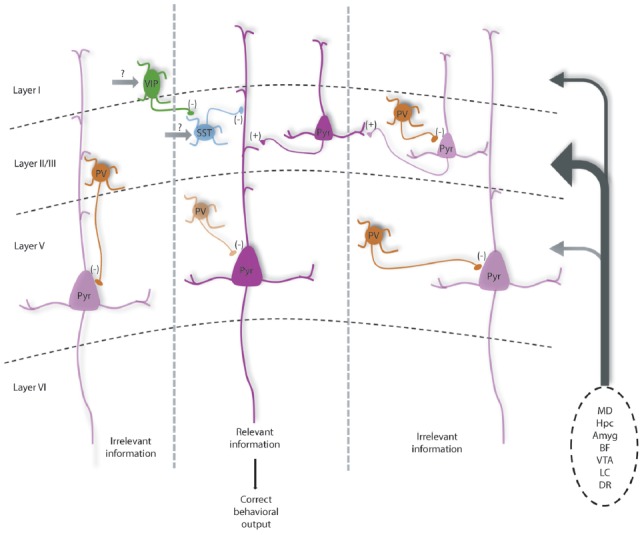
Diagram shows how parvalbumin (PV) interneurons help maintain the excitation and inhibition (E/I) balance within prefrontal local circuits for optimal information processing and how they are modulated by subcortical inputs. Under normal conditions, PV interneurons are highly active to help maintain a high level of inhibition relative to excitation in pyramidal neurons (potentially mediated by MD inputs). This may help dampen the activity of neurons in functional units representing distracting information (left and right cortical columns), increasing the signal to noise of incoming task-related information from prefrontal cortex (PFC) afferents (listed on right). PV interneurons likely help regulate spike timing as well as oscillatory patterns, which in concert would increase the likelihood that neurons representing relevant information are active and can relay correct information to downstream structures, driving the proper execution of PFC-dependent behaviors.Vasoactive-intestinal peptide (VIP) interneurons may help further refine cortical representations by inhibiting somatostatin (SST) neurons that target distal dendrites to disinhibit groups of excitatory pyramidal neurons. Darker and lighter shaded neurons represent high vs. low levels of activity respectively. This model is modified from Ferguson and Gao (2018) and is based on data discussed in the text and known connectivity within and projections to the medial PFC (mPFC; Kuroda et al., 2004; Rotaru et al., 2005; Hoover and Vertes, 2007). Ongoing questions involve what subcortical structures provide the majority of excitatory input to each neuronal subtype to regulate their function, as well as the precise contribution of recurrent excitation in regulating inhibitory neurons to affect E/I balance. Note: Amyg, amygdala; BF, basal forebrain; DR, dorsal raphe; Hpc, hippocampus; LC, locus coeruleus; MD, mediodorsal thalamus; VTA, ventral tegmental area.

In combination with numerous elegant studies from the visual, auditory and somatosensory systems, we can begin to develop a picture of how the mPFC microcircuit might be organized to represent and process information relevant for executive function. The largest source of afferents originates from the mediodorsal thalamus (MD), but mPFC neurons also receive convergent inputs from limbic structures including the hippocampus and amygdala, that may convey spatial and emotional information. Additionally, the mPFC has dense innervation from neuromodulatory regions, the ventral tegmental area (VTA), locus coeruleus (LC), dorsal raphe (DR) and basal forebrain (BF), which may influence the state of the animal including level of attention, arousal, or the salience of the current task or resulting rewards or punishment (Kuroda et al., 1996; Hoover and Vertes, 2007). Using working memory as an example, early studies in rodents found that neurons representing differential task-related information in a consistent manner were sparse (Jung et al., 1998, 2000; Baeg et al., 2003). However, more recent work has identified numerous mPFC neurons that change their firing frequency during distinct phases of a delayed-alternation task. Of these neurons, Yang et al. (2014) was able to separate these into units showing preferential firing on left vs. right trials, and encode information such as the choice, delay, and presence of reward. Other studies highlight a segregation of spatial and rule information encoding in mPFC neurons, of which the rule representation depends critically on the activity of the MD (Bolkan et al., 2017; Schmitt et al., 2017). Taken together, this suggests that pyramidal neurons in the mPFC form functional units that represent different features relevant to successful working memory performance and other PFC-dependent behaviors (Figure 1).

How might the appropriate E/I balance be maintained in the mPFC? As a general principle, thalamic inputs provide stronger activation to PV interneurons vs. pyramidal cells in sensory cortices (Hestrin, 1993; Kloc and Maffei, 2014). Similarly, enhancing excitability of MD neurons results in enhanced spiking of PV interneurons in mPFC, and not pyramidal cells (Schmitt et al., 2017), while dampening MD activity increases E/I balance by selectively reducing inhibitory currents onto pyramidal cells (Ferguson and Gao, 2018). Thus, the MD seems to function drive PV interneurons in the mPFC to maintain an E/I balance biased towards inhibition. Maintaining a network state with a high level of inhibition, would strengthen computational capacity through enhancing temporal precision and increasing the gain of pyramidal neuron responsiveness, as well as enhancing the signal to noise ratio of neurons encoding task or rule representations (Figure 1). Supporting that this large-scale inhibition is mainly PV interneuron mediated, we observed that enhancing PV interneuron excitability can fully normalize shifts in E/I balance with corresponding behavioral improvements (Ferguson and Gao, 2018).

PV interneurons are powerful regulators of pyramidal neuron activity and appear to be the most vulnerable across psychiatric disorders, but research highlights other major inhibitory interneurons that help fine-tune circuits supporting PFC-dependent behaviors (Wang et al., 2004). Axo-somatic inhibition accounts for only up to 10% of total inhibition received by excitatory neurons, while the remaining 90% occurs at proximal and distal dendrites and spines and is mediated by other inhibitory neuronal subtypes (Kubota et al., 2016). For example, SST interneurons exhibit phasic firing during distinct phases of a delayed alternation task, and seem to show segregation of firing among left vs. right trials (Kim D. et al., 2016). Further, in the hippocampus, SST interneurons provide powerful dendritic inhibition that helps to regulate both synaptic integration as well as the gain of pyramidal neuron responsiveness (Lovett-Barron et al., 2012). Additionally, vasoactive-intestinal peptide (VIP, 5HT3aR expressing) interneurons (Férézou et al., 2002), which inhibit both SST and PV interneurons seem to serve an important disinhibitory function in cortical circuits (Pi et al., 2013), that may help modulate the gain of pyramidal neuron responses (Fu et al., 2014). Recent data demonstrates VIP interneuron activity enhances mPFC circuit performance during two delay tasks involving working memory (Kamigaki and Dan, 2017). VIP interneurons may also facilitate forming associations in pyramidal neurons, as VIP interneurons show the greatest increase in activity along with pyramidal neurons in a go/no-go task following punishment (Pinto and Dan, 2015). As they inhibit both PV and SST cells, disinhibition of pyramidal cells by VIP interneurons may be a mechanism for facilitating the activity of neuronal ensembles relevant for optimal task performance in multiple PFC-dependent tasks (Figure 1). Further, targeting VIP interneuron activity has shown promising results in an animal model of hypofrontality in schizophrenia (Koukouli et al., 2017).

## Targeting E/I Balance in Animal Models to Improve Behavior

Multiple approaches for enhancing the normal function of prefrontal PV interneurons have been effective in mitigating a constellation of behavioral symptoms animal models of cognitive dysfunction and social abnormalities. For example, altering expression of Dlx5/6, a protein that regulates PV interneuron development leads to cognitive and other behavioral abnormalities in mice. Optogenetic activation of mPFC PV interneurons in Dlx5/6 mice is sufficient to ameliorate cognitive flexibility and social interaction deficits (Cho et al., 2015). Similarly, mice deficient in Contactin-associated protein-like (CNTNAP)–2 display marked social impairments that can be rescued by increasing PV interneuron activity optogenetically (Selimbeyoglu et al., 2017) in agreement with findings from naïve mice with optogenetically disrupted mPFC E/I balance (Yizhar et al., 2011). Dampening activity in the MD (modeling findings of mediodorsal hypofunction in schizophrenia) has been shown to impair working memory, cognitive flexibility, social interaction and alter anxiety-related behavior (Parnaudeau et al., 2013, 2015; Bolkan et al., 2017; Ferguson and Gao, 2018). However, pharmacogenetic activation of mPFC PV interneurons normalizes all mPFC-dependent behavioral deficits (Ferguson and Gao, 2018), highlighting the therapeutic potential of PV interneurons as a strategy for repairing E/I balance and treating a range of behavioral deficits. These results are complemented by pharmacological augmentation of GABAergic signaling, most commonly with benzodiazepines, that has been shown to repair behaviors including social interaction and cognitive flexibility (Wen et al., 2010; Han et al., 2012; Cho et al., 2015).

An ongoing question has been whether altering the properties of PV interneurons is responsible for the initial manifestation of behavioral deficits in psychiatric disease and animal models, in particular, those beyond the cognitive realm. For example altering the excitability of PV interneurons by genetic deletion of N-methyl-D-aspartate receptors (NMDARs), leads to selective deficits in working memory and associative learning (Carlen et al., 2012), while a broader deletion in various subtypes of cortical and hippocampal interneurons, induces cognitive impairments along with anxiety-related behavior, depressive symptoms and social impairments (Belforte et al., 2010). Similarly, reducing the expression of the voltage-gated sodium channel (Na_V_) 1.1, a channel linked to autism-related behaviors in Dravet syndrome, broadly in forebrain GABAergic interneurons induces spatial memory and social interaction deficits, along with increased repetitive behaviors and anxiety (Han et al., 2012). Optogenetic suppression of PV interneuron activity recapitulates only cognitive impairments, specifically a deficit in extradimensional set-shifting (Cho et al., 2015). Additionally, selective expression of tetanus toxin light chain (TeLC) in mPFC PV interneurons, disrupts working memory and cognitive flexibility, while sparing behaviors representing positive and negative symptoms in schizophrenia (Murray et al., 2015). However, in this study, recording of mPFC local field potentials revealed gamma oscillations did not differ between groups, so without further physiological analysis, the ultimate circuit consequences are somewhat ambiguous. Although it is unclear whether disrupting PV activity can disrupt a broader range of behaviors given different findings with articles employing disparate methodologies, the specificity of the insult seems to be critical. Solely targeting PV interneurons will reliably recapitulate phenotypes of cognitive disruption, while broader interneuronal insults increase the likelihood of impacting other behaviors. Another possibility is that the magnitude of the PV interneuron deficit may correlate directly with the propensity for disrupting behaviors beyond cognition.

## E/I Balance in Psychiatric Disease

The pattern of rodent PFC lesions and deficits mirrors the impairments observed following damage to the homologous structures in primates and humans, suggesting the functions of these brain regions are highly conserved across species. Even more compelling is how myriad findings recapitulate commonly observed morphological alterations and behavioral endophenotypes seen in psychiatric disease. One of the most demonstrative parallels is found in the pathophysiology and symptom sequelae of schizophrenia. Schizophrenia is a debilitating psychiatric disease that affects approximately 1.1% of the world’s population, and is characterized by positive, negative, and cognitive symptoms (Regier et al., 1993). Working memory and cognitive flexibility represent core dysfunctions in schizophrenia that remain intractable by treatment with current antipsychotics (Lee and Park, 2005; Insel, 2010). These along with a range of additional cognitive symptoms emerge prior to the onset of psychosis in early adulthood, and are the largest predictor of functional outcome in individual patients (Green, 1996). Negative symptoms involve anhedonia and social withdrawal, and are also minimally responsive to presently available treatments (Remington et al., 2016).

Analogous to experimental observations from animal studies, parallel deficits in PFC GABAergic signaling (Lewis et al., 2012), and E/I balance (Lisman, 2012) are both implicated in the pathophysiology of cognitive dysfunction in schizophrenia. Functional imaging reveals that schizophrenics exhibit patterns of hypofunction in the PFC (Van Snellenberg et al., 2016) as well as reduced volume in frontal cortex postmortem tissue (Selemon et al., 2002). Copious research demonstrates reductions in the marker for the GABA synthesizing enzyme, GAD-67, in PV interneurons in the PFC (Akbarian and Huang, 2006; Lewis et al., 2012). Levels of GAD-67 are activity-dependent (Benson et al., 1989; Sloviter et al., 1996), and coupled with the common finding that PV levels are also decreased within these interneurons (Glausier and Lewis, 2017), it suggests that the function of PFC PV interneurons is diminished in schizophrenia. Correspondingly, patients with schizophrenia show decreases in task-evoked in gamma oscillations, an oscillation band believed to be dependent on PV interneuronal firing, that correlate with the level of functional impairment in working memory (Basar-Eroglu et al., 2007).

Analogous to human pathology, rodent models of cognitive dysfunction in schizophrenia show an extremely high prevalence of reductions in PV or GAD67 expression in the mPFC. As more evidence reveals this disorder likely stems from heterogeneous etiologies, pharmacological, environmental, and transgenic models exhibit this common feature (Cochran et al., 2002; Francois et al., 2009; Carlson et al., 2011), suggesting that GABAergic hypofunction represents a convergence point. The constellation of data from human populations, animal models, experimental disruption of GABAergic signaling, and electrophysiological recordings across these contexts, highlight this system as a pathway to intervention in afflicted individuals.

Intriguingly, this pattern repeats among multiple psychiatric disorders including ASD, depression, and intellectual disability (Gao and Penzes, 2015; Luscher and Fuchs, 2015). ASD in particular harbors significant overlapping behavioral impairments and underlying neurobiological alterations. Autistic patients also exhibit prominent deficits in executive function, including working memory impairments and behavioral inflexibility (Hughes et al., 1994). Social abnormalities are also a behavioral hallmark of autistic pathology, primarily manifested as a deficit in non-verbal communication (Mundy et al., 1986). Autistic patients also suffer from a greater level of anxiety and depression relative to general population (Kim et al., 2000).

E/I balance disruption has also emerged as a prominent hypothesis in ASD (Rubenstein and Merzenich, 2003). Dysregulation of GABAergic signaling has been implicated in the etiology of ASD, and disorders sharing high comorbidity with the disease including anxiety and epilepsy (Coghlan et al., 2012). The chromosomal region 15q11-q13 is comprised of multiple genes encoding subunits of the GABA_A_R. Microduplications in this region (Cook et al., 1998; Menold et al., 2001) or aberrant expression of the associated gene products (Hogart et al., 2007; Mendez et al., 2013) have been frequently observed in ASD clinical populations. In addition to the finding of copy number variations in the 15qllq13 chromosomal locus, the majority of autism-linked genes are preferentially expressed in interneurons (Xu et al., 2014).

Supporting this are *in vivo* findings of reduced GABA and GABA_A_R levels in the frontal cortex of autistic patients (Harada et al., 2011; Mori et al., 2012), along with reductions in gamma oscillations (Sun et al., 2012). Similar deficiencies in inhibitory neurotransmission have been reported in mice with mutations in ASD-linked genes, mice that also exhibit relevant behavioral impairments (Peñagarikano et al., 2011; Han et al., 2012). For example, T(+)Itpr3(tf)/J (BTBR) mice, a model of idiopathic autism, exhibit decreased GABAergic currents and increased excitatory neurotransmission in the hippocampus, indicating a shift in the E/I balance. Concurrent with these physiological changes, these mice display reductions in sociability, cognitive impairments, and alterations in anxiety-related behaviors (Han et al., 2014). Additionally, mouse models with disruption of genes such as methyl-CpG-binding protein-2 (MECP2), Scn1a^+/–^ and CNTNAP–2, all harbor interneuron deficits along with behavioral impairments (Chao et al., 2010; Peñagarikano et al., 2011; Han et al., 2012). Modeling of Fragile X syndrome, a genetic disorder associated with intellectual disability, also reveals cognitive impairments are associated with prominent GABAergic hypofunction in mice (Selby et al., 2007; Curia et al., 2009).

## GABAergic Signaling as a Therapeutic Target

Multiple studies suggest that augmenting GABAergic signaling via PV interneuron modulation can be effective in ameliorating deficits in working memory, cognitive flexibility and sociability in animal models of psychiatric disease. This has substantial implications for schizophrenia, given that cognitive symptoms are treatment-resistant with both typical and atypical antipsychotics, and are a significant predictor of quality of life in individual patients (Green, 2006). These results complement a rich history of data and hypotheses surrounding GABAergic hypofunction in the human PFC representing a final common pathway in the cognitive symptoms in schizophrenia (Lewis, 2014). Yet, we still await the discovery of a GABAergic modulator that displays effectiveness in alleviating cognitive dysfunction across multiple disorders.

Of the recent clinical trials targeting cognitive symptoms, very few utilize GABAergic drugs, relative to agonists and modulators of glutamatergic signaling. Although benzodiazepines have shown potential in treating psychosis (Wolkowitz and Pickar, 1991; Carpenter et al., 1999), their potential in reducing cognitive impairments remains largely unexplored with the exception of a few studies. Interestingly in one study, a benzodiazepine, lorazepam, exacerbated working memory impairments in schizophrenic patients and healthy controls and altered activity within networks supporting cognition. Researchers concluded that hyper-inhibition was responsible for cognitive dysfunction, and correspondingly, flumazenil an antagonist of the benzodiazepine site attenuated deficits (Menzies et al., 2007). However, a separate study found iomazenil, a flumazenil analog, increased psychoses in schizophrenic patients (Ahn et al., 2011). Given the high comorbidity of ASD with epilepsy (Canitano, 2007), researchers have gleaned that drugs effective in reducing seizure activity that typically works through elevating GABA levels also mitigate autistic symptoms (Jambaqué et al., 2000). Promisingly, GABA agonists have reached Phase II clinical trials for the treatment of social disability in ASD, but so far have only focused on α2/α3-GABA_A_Rs.

The synapse between PV-expressing chandelier cells and the axon initial segment have become frequent but unsuccessful target for therapeutics in schizophrenia. In patients with schizophrenia, chandelier cell axon terminals exhibit decreased levels of the reuptake enzyme, GABA transporter 1 (GAT1) mRNA, along with higher expression of α2-GABA_A_Rs on the axon initial segment of pyramidal neurons (Volk et al., 2002). These alterations would result in less GABA reuptake, and increased post-synaptic GABAergic inhibition respectively, which together would serve to augment GABAergic neurotransmission at these particular synapses. This has been interpreted as a compensatory mechanism, implying these synapses may be the site of the initial GABAergic deficit (Volk and Lewis, 2005). However, despite promising results from early studies (Lewis et al., 2008), larger sample sizes have yielded no differences between schizophrenic patients treated with an α2/α3-GABA_A_R agonists compared to those treated with the placebo (Buchanan et al., 2011). Given the failure of enhancing α2-GABA_A_R-mediated signaling in improving cognitive dysfunction in clinical trials as well as in the context of compensatory mechanisms in schizophrenia, it is reasonable to conclude these receptors are not a viable therapeutic target.

One difficulty in utilizing GABAergic therapeutics, including benzodiazepines, is their sedative properties, which are mediated by their action at the α1 GABA_A_R subunit (Löw et al., 2000). This may account for the lack of exploration of α1-GABA_A_R modulators in clinical trials. However the actions of dopamine across different subjects and contexts provide a compelling example of how optimal levels of neurotransmitter can be associated with normal cognition, attention and alertness, but levels either too high or low, can result in impaired cognition, inability to focus, and drowsiness (Cools and D’Esposito, 2011). Our findings using indiplon, an α1-GABA_A_R positive allosteric modulator (Ferguson and Gao, 2018), and other studies using benzodiazepines (Han et al., 2012, 2014) suggest that the therapeutic window for influencing cognitive function may be distinct from doses that induce sedation. It is likely that GABAergic signaling in the PFC and its correlated functions follow an inverted-U trajectory, and with better biomarkers for individual differences in GABA levels, indiplon, novel α1-GABA_A_R modulators or benzodiazepines may harbor significant therapeutic potential.

## Future Directions

Additional questions remain that could help inform a circuit model of information processing in the mPFC. For example, how organized are the actions of PV interneurons in the mPFC? Are they providing a general blanket inhibition that helps maintain a cortical state conducive to information processing due to their properties described above? Evidence supporting this is imaging of PV interneurons revealing that PV interneurons show the largest modulation, and have uniform firing patterns that persist throughout delay periods during PFC-dependent tasks in comparison to other interneuron subtypes (Pinto and Dan, 2015; Kim D. et al., 2016). If PV interneurons do provide indiscriminate inhibition throughout the mPFC, to what extent to disinhibitory circuits through VIP and SST interneurons help further enhance information representation, and through what mechanisms and inputs? Other data indicate segregated groups of PV interneurons encode distinct task phases (Lagler et al., 2016), suggesting instead of blanket inhibition, patterns of activity are differentially regulated across different PV interneurons. Future studies should also further explore the actions of PV chandelier cells in the mPFC during behavior. New research indicates this subtype may preferentially inhibit amygdala-projecting pyramidal neurons in the mPFC, suggesting chandelier cells have a distinct role in the mPFC microcircuit from PV basket cells (Lu et al., 2017).

Currently available imaging and physiological methods provide promising approaches for measuring changes in neuronal activity that are correlated with successful behavior. However, given the diversity of neocortical interneuron subtypes and function, how PV interneurons and other cell types contribute to modulation of E/I balance, and whether and how modulation of each subtype impact different PFC-dependent behaviors warrants further investigation. We still lack effective treatments for cognitive deficits, social interaction impairments, and other associated behavioral dysfunctions in numerous psychiatric disorders, underscoring the importance of continuing to unravel how these behavioral processes occur under normal conditions. If researchers can identify how to effectively manipulate PFC circuit activity, we can develop reliable strategies for engineering optimal patterns of cortical activity to ameliorate performance. Ultimately, if successful manipulations can be linked to physiological signatures that can be observed using less invasive recording approaches such as oscillatory patterns, we can potentially determine biomarkers for successful behavioral therapies in humans.

## Author Contributions

BF wrote the manuscript and W-JG edited it.

## Conflict of Interest Statement

The authors declare that the research was conducted in the absence of any commercial or financial relationships that could be construed as a potential conflict of interest.
